# CD52 Is a Prognostic Biomarker and Associated With Tumor Microenvironment in Breast Cancer

**DOI:** 10.3389/fgene.2020.578002

**Published:** 2020-11-02

**Authors:** Jianxin Wang, Guangchen Zhang, Yang Sui, Zhuowen Yang, Yinzhu Chu, Hailing Tang, Binbin Guo, Cong Zhang, Changjun Wu

**Affiliations:** ^1^Department of Ultrasound, The First Affiliated Hospital of Harbin Medical University, Harbin, China; ^2^Department of Cardiology, The First Affiliated Hospital of Harbin Medical University, Harbin, China

**Keywords:** breast cancer, CD52, tumor microenvironment, immune infiltration, prognosis

## Abstract

Tumor microenvironment (TME) plays an essential role in the development and metastasis of breast cancer (BC). More studies are needed on the differences and functions of immune components and matrix components. In this study, we calculated the proportion of tumor-infiltrating immune cells (TICs) and the proportion of immune and matrix components of BC patients from The Cancer Genome Atlas (TCGA). We performed Cox regression analysis and constructed protein-protein interaction (PPI) network based on the differentially expressed genes (DEGs) and obtained the most crucial gene *CD52*. CD52 significantly upregulated and affected the prognosis of BC patients. Gene set enrichment analysis (GSEA) suggested that the genes in the CD52 high-expression group were mainly enriched in immune-related pathways, while those in the CD52 low-expression group were mainly enriched in metabolic pathways. TICs analyses showed that there should be a positive correlation between CD52 expression and CD8+ T cells, activated memory CD4+ T cells, macrophage M1, and Gamma Delta T cells. It indicated that CD52 might be an essential factor in maintaining the immune-dominant position of TME. These results suggest that CD52 might be a potential biomarker for prognosis and provide a new therapeutic target for BC patients.

## Introduction

Breast cancer (BC) is one of the most common tumors diagnosed by women. According to the American Cancer Association’s latest statistics, BC patients account for 30% of all female cancer patients, and the mortality rate is the highest among 20–59-year-old female cancer patients (Siegel et al., 2020). With the popularization of mammography, BC’s early diagnosis rate has increased, and the mortality rate has declined. However, in recent years, BC mortality’s declining trend is not optimistic (DeSantis et al., 2019). For BC patients, chemotherapy is the most used treatment. In the past 30 years, the research and application of targeted therapy have improved the survival rate of metastatic breast cancer (Jin et al., 2018). However, the research progress of targeted treatment for breast cancer types without precise biomarkers is relatively backward, and it still stops at the stage of non-metastatic breast cancer treatment (Waks and Winer, 2019). Besides, the emergence of patients’ drug resistance to chemotherapy drugs makes the therapeutic effect of existing targeted drugs hit (Chen and Zhang, 2018; Yang et al., 2019). Therefore, the discovery and development of more extensive tumor markers and new targeted drugs are the bottlenecks of targeted therapy.

The tumor microenvironment (TME) is an essential factor that affects tumor behavior, in which immune cells play an essential role (Gajewski et al., 2013). The characteristics of the immune microenvironment changed dynamically with tumor progression. BC is a kind of tumor characterized by an inflammatory response, and the immune cells are abundant in the microenvironment (Tower et al., 2019). More and more studies show that infiltrating immune cells in the TME can be the target of treatment and the target of therapeutic effect. Especially, tumor-infiltrating lymphocytes have been proved to be related to the good response and better prognosis of chemotherapy (Pruneri et al., 2016). In neoadjuvant chemotherapy, the presence of tumor-infiltrating lymphocytes is associated with a high pathological response rate (Denkert et al., 2010). Given the PD-1/PD-L1 pathway that induces immune escape between tumor and T lymphocyte, the advent of the PD-1 inhibitor undoubtedly provides a new possibility for targeted therapy (Bastaki et al., 2020). From these results, immune cells have very subtle functional transformation under the regulation of tumor cells, and the state of activation or inhibition of immune cells also affects the survival state of the tumor. Therefore, it is crucial to determine the factors that affect the dynamic changes of immune cells at the gene level for targeted BC treatment.

In this study, we calculated the proportion of tumor-infiltrating immune cells (TICs) and the proportion of immune and matrix components of BC samples from The Cancer Genome Atlas (TCGA) database and determined a useful predictive biomarker CD52. CD52 is a membrane glycoprotein widely expressed on the surface of mature lymphocytes, monocytes, and dendritic cells. The monoclonal antibody Alemtuzumab combined with CD52 is commonly used in treating chronic lymphoblastic leukemia and multiple sclerosis, but its role in solid tumors has not been studied (Badoux et al., 2011; Cohen et al., 2012; Zhao et al., 2017). Therefore, CD52 might be an unexplored biomarker related to immune cell regulation in BC.

## Materials and Methods

### Data Source

We obtained RNA-sequence of 1222 BC samples (1,109 tumor samples and 113 healthy samples) and clinical information from the TCGA database.^1^

### Calculation of ImmuneScore and StromalScore

We used the ESTIMATE algorithm with the “estimate” package in R to calculate the proportion of immune and matrix components in the TME of each sample, embodied in ImmuneScore and StromalScore. We combined ImmuneScore and StromalScore with follow-up information on BC patients for survival analysis. A value of *p* < 0.05 was considered significant.

### Identification of DEGs

The BC samples were labeled high or low based on median scores compared with ImmuneScore and StromalScore. The “limma” R package was used to identify differentially expressed genes (DEGs) between the high group and low group according to the cut-off criteria of |log 2 FC| > 1 and false discovery rate (FDR) < 0.05 (Ritchie et al., 2015).

### GO and KEGG Terms Enrichment Analysis

To explore the functional correlation of these sharing DEGs, we used the “clusterProfiler” R package to perform Gene Ontology (GO) functional annotations and Kyoto Encyclopedia of Genes and Genomes (KEGG) pathway enrichment analysis (Yu et al., 2012). Those with *p*‐ and *q*-values < 0.05 were considered as significant categories.

### PPI Network Construction

To further explore its potential mechanism, we built a protein-protein interaction (PPI) network based on the String database^2^ using Cytoscape’s software (version 3.8.0). Nodes with interaction confidence greater than 0.70 were used to construct the network.

### Univariate Cox Regression Analysis

We performed univariate Cox regression analysis to identify the DEGs associated with overall survival (OS). Select DEGs with a value of *p* < 0.01 for further analysis. Intersecting with the results of PPI, we got the gene *CD52* for further study.

### Differential Expression and Survival Analysis of CD52

We verified the difference of CD52 expression between tumor and normal samples by the Wilcoxon rank-sum test. We also analyzed the CD52 expression in paired samples of normal and tumor tissues of the same patient. Kaplan–Meier (KM) method was used to analyze the effect of CD52 on the survival of BC patients. We downloaded the METABRIC cohort of breast cancer patients and combined CD52 expression with clinical follow-up data, including 1,904 patients for survival analysis.

### Gene Set Enrichment Analysis

We performed gene set enrichment analysis (GSEA) with GSEA software.^3^ The enrichment score (ES) > 0.4 as a filter and FDR value < 0.05 were statistically significant.

### CIBERSORT Analysis

To calculate the relationship between CD52 expression and the TICs abundance distribution of all BC samples, we used the CIBERSORT algorithm to estimate the relative abundance of 22 types of infiltrating immune cells, including macrophages, T cells, B cells, and other immune cells (Newman et al., 2015). A value of *p* < 0.05 was set as the threshold, and the CIBERSORT output was analyzed to determine the difference between TICs and CD52 expressions.

## Results

### Scores Correlated With Survival of BC Patients

**Figure 1:**
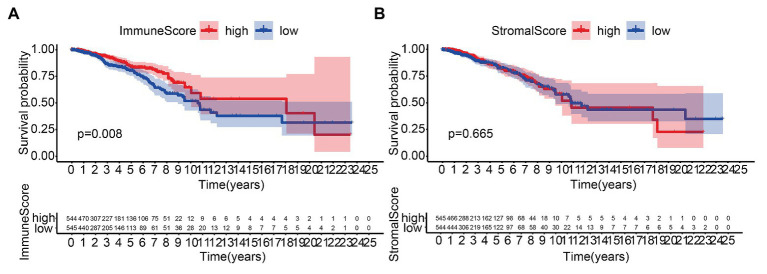
Correlation of scores with the survival of patients with breast cancer (BC). **(A)** Kaplan–Meier (KM) survival analysis for BC patients grouped into the high or low score in ImmuneScore determined by comparing the median. **(B)** Kaplan–Meier survival curve for StromalScore.

We performed the KM analysis by combining ImmuneScore and StromalScore with survival time and state of patients. The proportion of immune components was significantly related to the OS of BC patients (Figure 1A), while the proportion of matrix components was not significantly correlated to the OS (Figure 1B).

### DEGs Screening

**Figure 2:**
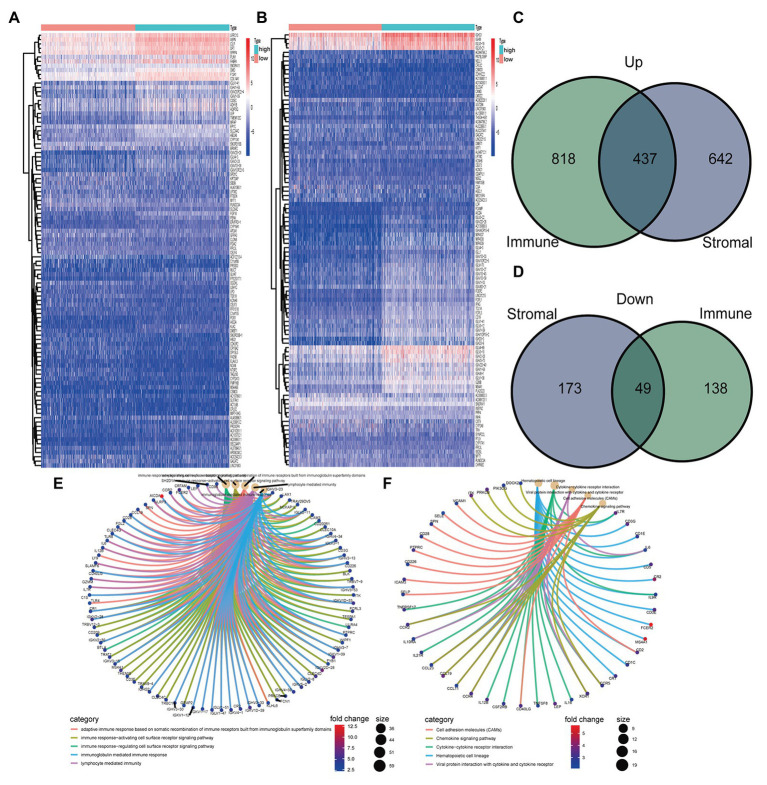
Heatmaps, Venn plots, and enrichment analysis of gene ontology (GO) and Kyoto Encyclopedia of Genes and Genomes (KEGG) for differentially expressed genes (DEGs). **(A)** Heatmap for DEGs generated in StromalScore. **(B)** Heatmap for DEGs in ImmuneScore. **(C,D)** Venn plots were showing common upregulated and downregulated DEGs shared by ImmuneScore and StromalScore. **(E,F)** GO and KEGG enrichment analyses for DEGs

One thousand three hundred and one genes were identified from StromalScore, including 1,079 upregulated genes and 222 downregulated genes (Figure 2A). One thousand four hundred and forty-two DEGs were identified from ImmuneScore, consisting of 1,255 upregulated genes and 187 downregulated genes (Figure 2B). We obtained 437 upregulated genes and 49 downregulated from the intersection analysis (Figures 2C,D).

### Functional Enrichment Analysis

Gene ontology analysis showed that DEGs were mainly enriched in immune-related functions, including adaptive immune response based on somatic recombination of immune receptors built from immunoglobulin superfamily domains, immune response-activating cell surface receptor signaling pathway, immune response-regulating cell surface receptor signaling pathway, immunoglobulin mediated immune response, and lymphocyte-mediated immunity (Figure 2E). Similarly, KEGG analysis showed that DEGs were mainly enriched in immune-related pathways, including cell adhesion molecules, chemokine signaling pathway, cytokine-cytokine receptor interaction, hematopoietic cell lineage, and viral protein interaction with cytokine and cytokine receptor (Figure 2F).

### Hub Gene Identification and Cox Regression Analysis

**Figure 3:**
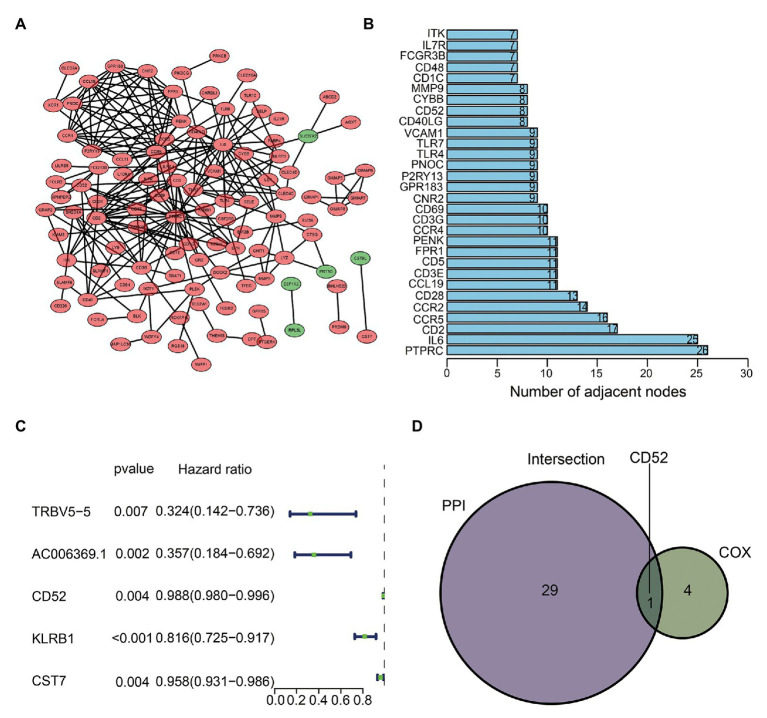
Protein-protein interaction (PPI) network and Cox regression analysis. **(A)** Construction of interaction network. **(B)** The number of nodes orders the top 30 genes. **(C)** Forest plot of Cox regression analysis. **(D)** Venn plot showing the sharing factors by the top 30 genes in PPI and significant genes in Cox.

We used sharing DEGs to construct a PPI network by Cytoscape (Figure 3A). The top 30 genes with the most nodes in the PPI network were identified as the hub genes (Figure 3B). Cox regression analysis showed that five genes (*TRBV5-5*, *AC006369.1*, *CD52*, *KLRB1*, and *CST7*) were closely related to BC’s OS (Figure 3C). The intersection analysis PPI and Cox results obtained the most critical gene *CD52* (Figure 3D).

### CD52 Expression and Survival Analysis

**Figure 4:**
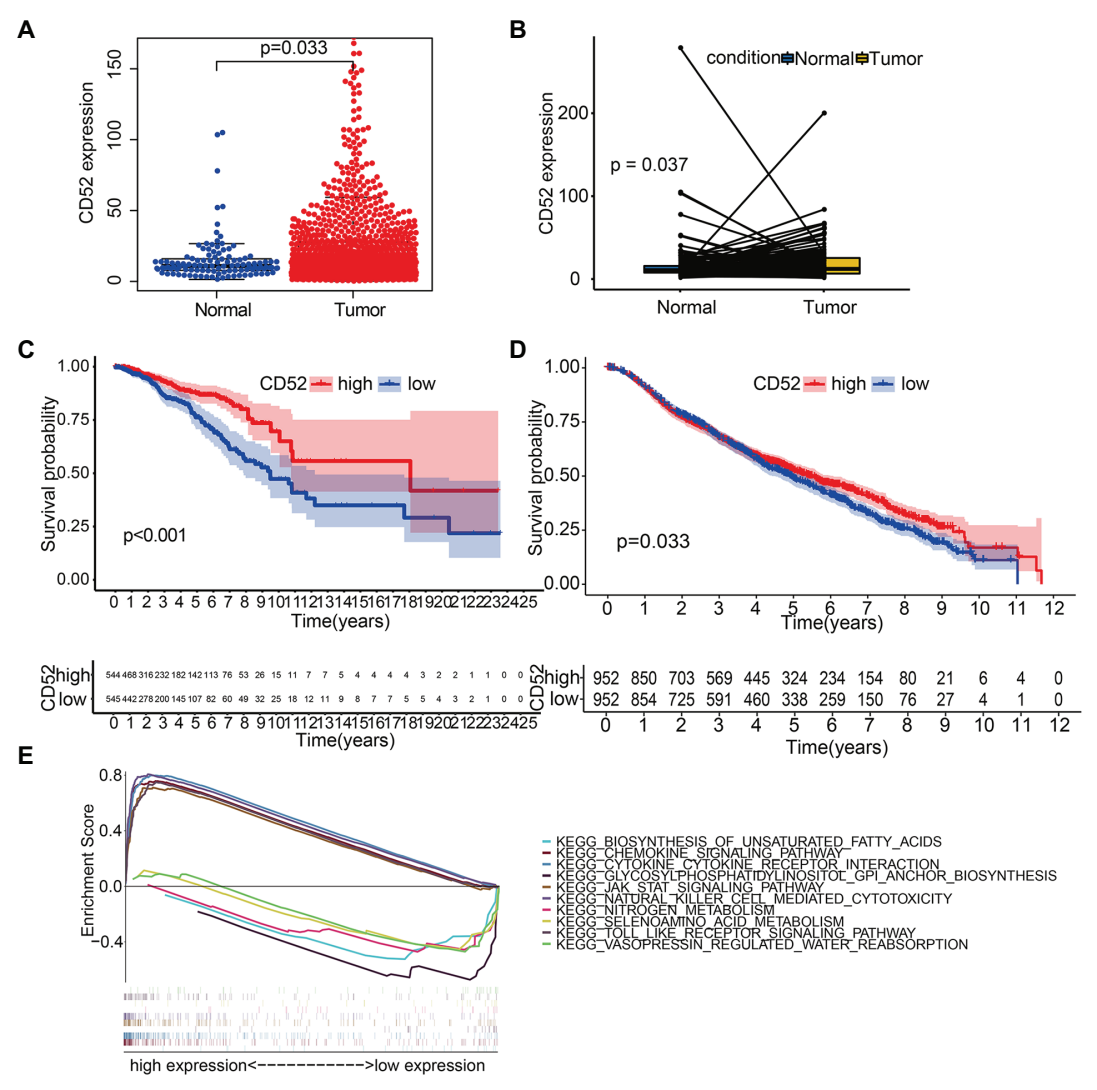
The differentiated expression of CD52, correlation with the survival of BC patients, and gene set enrichment analysis (GSEA). **(A)** Differentiated expression of CD52 in the normal and BC samples. **(B)** Paired differentiation analysis for expression of CD52. **(C)** Survival analysis for BC patients with different CD52 expressions in The Cancer Genome Atlas (TCGA) database. **(D)** Survival analysis for BC patients with different CD52 expressions in the METABRIC cohort. **(E)** The top five significant pathways of high and low expressions, respectively.

We found that CD52 was significantly upregulated in BC samples (Figure 4A), and the same results were observed in the paired samples (Figure 4B). Survival analysis showed that CD52 had an excellent ability to predict BC patients’ prognosis in the TCGA database (Figure 4C, *p* < 0.001). The METABRIC cohort also verified the prognostic value of CD52 (Figure 4D, *p* = 0.033).

### Gene Set Enrichment Analysis

Gene set enrichment analysis (GSEA) suggested that the genes in the CD52 high expression group were mainly enriched in immune-related pathways, while those in the CD52 low expression group were mainly enriched in metabolic pathways (Figure 4E).

### Correlation Between CD52 and TICs

**Figure 5:**
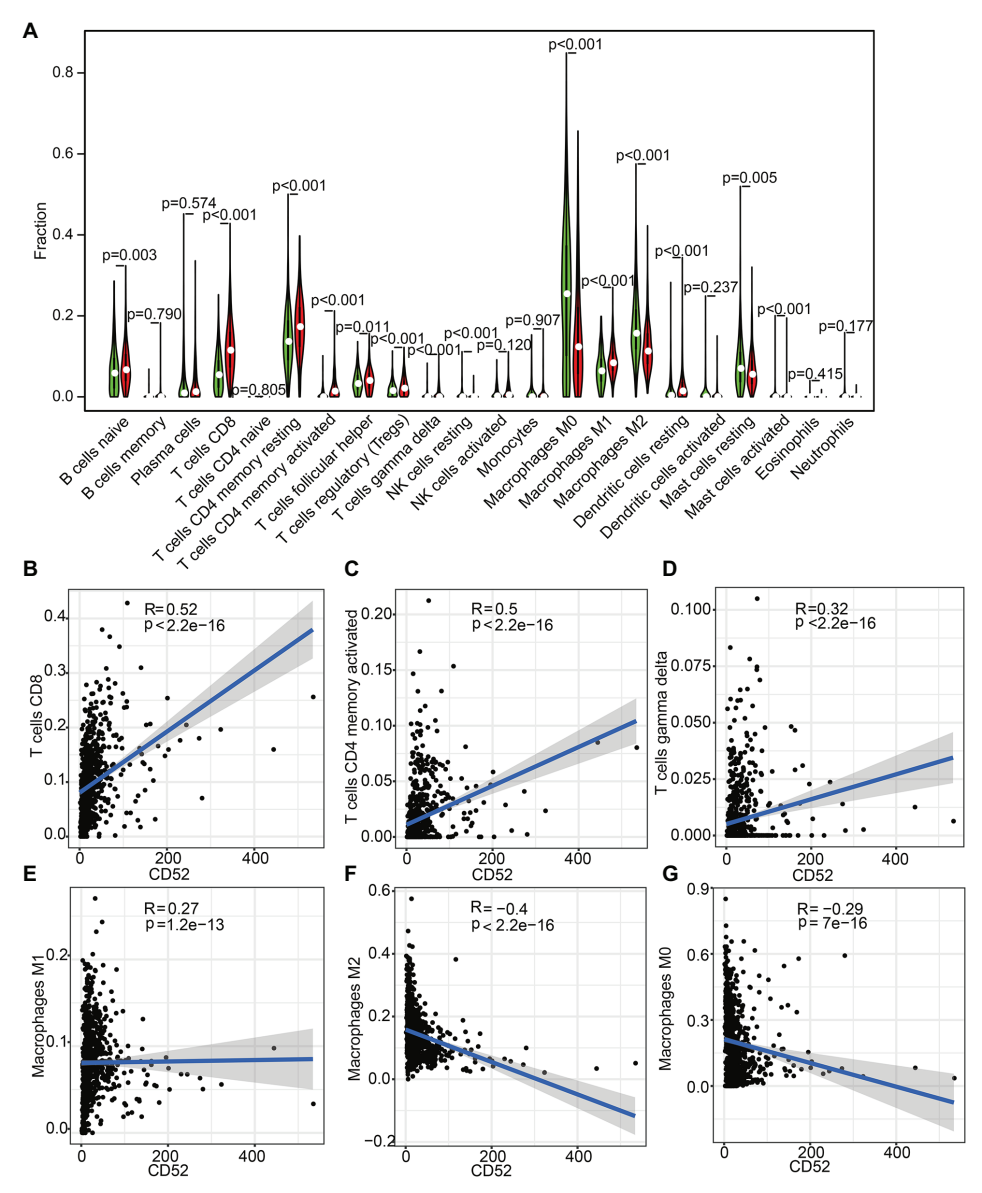
Tumor-infiltrating immune cell (TIC) profile in tumor samples and correlation analysis. **(A)** The Violin plot showed a correlation between CD52 and TICs. **(B–G)** The Scatter plot showed the Pearson’s correlation of the most significant cell infiltration with the CD52 expression.

To further explore the correlation between CD52 expression and immune microenvironment, we constructed the relative abundance of 22 types of infiltrating immune cells in BC samples. It had a significant correlation with 14 kinds of immune cells (Figure 5A). Among them, there should be a positive correlation between CD52 expression and CD8+ T cells, activated memory CD4+ T cells, macrophage M1, and Gamma Delta T cells (Figures 5B–E). There should be a negative correlation between CD52 expression and macrophages M0 and M2 (Figures 5F,G).

## Discussion

The TME has been proved to play an essential role in the occurrence, development, and metastasis of tumors. Especially the immune cells infiltrated in the TME, under the regulation of tumor, the behavior of immune cells is not coordinated, and the activation state and function of different immune cells incline to the direction of anti-tumor or promoting tumor under the regulation of tumor (Tower et al., 2019). Therefore, it is crucial to find the critical markers of regulating tumor immune microenvironment for reversing TME and promoting tumor state.

CD52 is a glycoprotein composed of 12 amino acids and an amino acid terminal oligosaccharide linked with asparagine. It is anchored on the cell membrane by a glycosylphosphatidylinositol (GPI) and widely expressed in mature lymphocytes, monocytes, dendritic cells, and NK cells (Treumann et al., 1995; Hale, 2001a b). Its targeted Alemtuzumab can induce cell lysis by complement to induce apoptosis of immune cells to achieve immunosuppression. It is also used as a high immunosuppressive drug in chronic lymphoblastic leukemia, lymphoma, multiple sclerosis, and other autoimmune diseases (Boyd and Dearden, 2008; Winqvist et al., 2017; Zmira et al., 2020). In recent years, the role of soluble CD52 has been discussed. Under the stimulation of glutamic acid decarboxylase, T lymphocytes with high expression of CD52 hydrolyze GPI under the action of phospholipase C, which leads to the release of soluble CD52. Soluble CD52 binds to sialic acid-binding immunoglobulin-like lectin-10 (Siglec10), a signal molecule of immunosuppression on the surface of T cells, which inhibits the activation and proliferation of immune cells, especially CD4+ T cells, and does the immune body damage caused by over activation of the immune system (Bandala-Sanchez et al., 2013). In addition to its inhibitory effect on T cell activation, soluble CD52 can also inhibit the toll-like receptor and tumor necrosis factor receptor pathways, inhibit the production of NF-kB, and the production of pro-inflammatory factors (Rashidi et al., 2018). However, the role of CD52 as a cell membrane surface receptor and in TME is not yet clear.

In this study, we identified that CD52 might play an important role in tumor immunity by regulating the TME of BC. GSEA analysis found that the immune response-related pathways in the CD52 high expression group were significantly enriched, such as cytokine and chemokine action pathway, T-cell receptor and B-cell receptor NK cell-mediated cytotoxicity pathway, and toll-like receptor-mediated immune activation pathway. In contrast, GPI synthesis, glucose metabolism, lipid metabolism, and other pathways were enriched in the CD52 low expression group. The results suggested that the expression and microenvironment of CD52 changed from immune response to metabolism. The regulatory mechanism of CD52 gene expression has not been clarified. Little is known about the role of CD52 as a membrane surface molecule. In the past studies, it was found that CD52 was highly expressed on the surface of T cells induced by quiescence and low expressed on T cells in the dividing stage. Many people think that CD52 is a sign of relatively disabled T cells (Kubota et al., 1990; Haaland et al., 2005). In the treatment of chronic lymphocytic leukemia, Alemtuzumab recognizes T cells with high expression of CD52, eliminates the disabled T cells through cell lysis, and restores the normal immune function of T cells (Bandala-Sanchez et al., 2013). In our study, we found that the high expression of CD52 is related to a good prognosis and a good survival rate, which may be the reason why CD52 regulates the maladjusted immune state. CD52 may play a dual role in the microenvironment of solid tumors, while CD52 falls off from the surface of the cell membrane to become soluble CD52, which makes CD52 change from a two-way regulator to a one-way regulator.

Using the algorithms of ESTIMATE and CIBERSORT and analyzing the gene enrichment of BC in the TCGA database, we determined that CD52 was related to the prognosis of BC patients. CD52 can be used as a biomarker and regulator of immune status in the TME. Based on the results of enrichment analysis and tic analysis, the immunomodulatory effect of CD52 in the tumor environment was speculated. The regulatory mechanism of CD52 gene expression, the regulatory pathway of CD52 as a surface receptor, and the role of CD52 on immune cells other than T cells in the solid TME need to be explored and studied urgently.

## Conclusion

In conclusion, our results suggested that CD52 might affect the prognosis of BC through its involvement in immune activity in TME. CD52 might be a biomarker to predict the immune response of the TME and provide a new therapeutic target for BC patients.

## Data Availability Statement

The datasets are available from TCGA (https://portal.gdc.cancer.gov/) and Gene Expression Omnibus (https://www.ncbi.nlm.nih.gov/geo/).

## Author Contributions

JW, GZ, and YS wrote the manuscript. JW, GZ, ZY, YC, and CZ analyzed data. HT, BG, and YC were responsible for the acquisition and interpretation of data. CW designed the research and revised the manuscript. All authors contributed to the article and approved the submitted version.

### Conflict of Interest

The authors declare that the research was conducted in the absence of any commercial or financial relationships that could be construed as a potential conflict of interest.

## Footnotes

^1^https://portal.gdc.cancer.gov/

^2^https://string-db.org/

^3^https://www.gsea-msigdb.org/gsea/index.jsp

## Abbreviations

BC: Breast cancer
DEGs: Differentially expressed genes
ES: Enrichment score
GO: Gene ontology
FDR: False discovery rate
GPI: Glycosylphosphatidylinositol
GSEA: Gene set enrichment analysis
KEGG: Kyoto Encyclopedia of Gene and Genome
KM: Kaplan–Meier
OS: Overall survival
PPI: Protein-protein interaction
ROC: Receiver operating characteristic
TCGA: The Cancer Genome Atlas
TICs: Tumor-infiltrating immune cells
TME: Tumor microenvironment

## References

[ref1] Badoux X. C. Keating M. J. Wang X. O’Brien S. M. Ferrajoli A. Faderl S.. (2011). Cyclophosphamide, fludarabine, alemtuzumab, and rituximab as salvage therapy for heavily pretreated patients with chronic lymphocytic leukemia. Blood 118, 2085–2093. 10.1182/blood-2011-03-341032, PMID: 21670470 PMC4123326

[ref2] Bandala-Sanchez E. Zhang Y. Reinwald S. Dromey J. A. Lee B. H. Qian J.. (2013). T cell regulation mediated by interaction of soluble CD52 with the inhibitory receptor Siglec-10. Nat. Immunol. 14, 741–748. 10.1038/ni.2610, PMID: 23685786

[ref3] Bastaki S. Irandoust M. Ahmadi A. Hojjat-Farsangi M. Ambrose P. Hallaj S.. (2020). PD-L1/PD-1 axis as a potent therapeutic target in breast cancer. Life Sci. 247:117437. 10.1016/j.lfs.2020.117437, PMID: 32070710

[ref4] Boyd K. Dearden C. E. (2008). Alemtuzumab in the treatment of chronic lymphocytic lymphoma. Expert. Rev. Anticancer. Ther. 8, 525–533. 10.1586/14737140.8.4.525, PMID: 18402519

[ref5] Chen Y. Zhang Y. (2018). Application of the CRISPR/Cas9 system to drug resistance in breast cancer. Adv. Sci. 5:1700964. 10.1002/advs.201700964, PMID: 29938175 PMC6010891

[ref6] Cohen J. A. Coles A. J. Arnold D. L. Confavreux C. Fox E. J. Hartung H. P.. (2012). Alemtuzumab versus interferon beta 1a as first-line treatment for patients with relapsing-remitting multiple sclerosis: a randomised controlled phase 3 trial. Lancet 380, 1819–1828. 10.1016/S0140-6736(12)61769-3, PMID: 23122652

[ref7] Denkert C. Loibl S. Noske A. Roller M. Müller B. M. Komor M.. (2010). Tumor-associated lymphocytes as an independent predictor of response to neoadjuvant chemotherapy in breast cancer. J. Clin. Oncol. 28, 105–113. 10.1200/JCO.2009.23.7370, PMID: 19917869

[ref8] DeSantis C. E. Ma J. Gaudet M. M. Newman L. A. Miller K. D. Sauer A. G. (2019). Breast cancer statistics, 2019. CA Cancer J. Clin. 69, 438–451. 10.3322/caac.21583 31577379

[ref9] Gajewski T. F. Schreiber H. Fu Y. X. (2013). Innate and adaptive immune cells in the tumor microenvironment. Nat. Immunol. 14, 1014–1022. 10.1038/ni.2703, PMID: 24048123 PMC4118725

[ref10] Haaland R. E. Yu W. Rice A. P. (2005). Identification of LKLF-regulated genes in quiescent CD4+ T lymphocytes. Mol. Immunol. 42, 627–641. 10.1016/j.molimm.2004.09.012, PMID: 15607822

[ref11] Hale G. (2001a). Cd52 (Campath1). J. Biol. Regul. Homeost. Agents 15, 386–391. PMID: 11860230

[ref12] Hale G. (2001b). The CD52 antigen and development of the CAMPATH antibodies. Cytotherapy 3, 137–143. 10.1080/146532401753174098, PMID: 12171721

[ref13] Jin J. L. Plevritis S. K. Tian L. Cadham C. J. Xu C. Stout N. K.. (2018). Change in survival in metastatic breast cancer with treatment advances: meta-analysis and systematic review. JNCI Cancer Spectr. 2:pky062. 10.1093/jncics/pky062, PMID: 30627694 PMC6305243

[ref14] Kubota H. Okazaki H. Onuma M. Kano S. Hattori M. Minato N. (1990). Identification and gene cloning of a new phosphatidylinositol-linked antigen expressed on mature lymphocytes. Down-regulation by lymphocyte activation. J. Immunol. 145, 3924–3931. PMID: 2147207

[ref15] Newman A. M. Liu C. L. Green M. R. Gentles A. J. Feng W. Xu Y.. (2015). Robust enumeration of cell subsets from tissue expression profiles. Nat. Methods 12, 453–457. 10.1038/nmeth.3337, PMID: 25822800 PMC4739640

[ref16] Pruneri G. Gray K. P. Vingiani A. Viale G. Curigliano G. Criscitiello C.. (2016). Tumor-infiltrating lymphocytes (TILs) are a powerful prognostic marker in patients with triple-negative breast cancer enrolled in the IBCSG phase III randomized clinical trial 22-00. Breast Cancer Res. Treat. 158, 323–331. 10.1007/s10549-016-3863-3, PMID: 27372069 PMC4977583

[ref17] Rashidi M. Bandala-Sanchez E. Lawlor K. E. Zhang Y. Neale A. M. Vijayaraj S. L.. (2018). CD52 inhibits toll-like receptor activation of NF-kappaB and triggers apoptosis to suppress inflammation. Cell Death Differ. 25, 392–405. 10.1038/cdd.2017.173, PMID: 29244050 PMC5762852

[ref18] Ritchie M. E. Phipson B. Wu D. Hu Y. Law C. W. Shi W.. (2015). Limma powers differential expression analyses for RNA-sequencing and microarray studies. Nucleic Acids Res. 43:e47. 10.1093/nar/gkv007, PMID: 25605792 PMC4402510

[ref19] Siegel R. L. Miller K. D. Jemal A. (2020). Cancer statistics, 2020. CA Cancer J. Clin. 70, 7–30. 10.3322/caac.21590, PMID: 31912902

[ref20] Tower H. Ruppert M. Britt K. (2019). The immune microenvironment of breast cancer progression. Cancer 11:1375. 10.3390/cancers11091375, PMID: 31527531 PMC6769749

[ref21] Treumann A. Lifely M. R. Schneider P. Ferguson M. A. (1995). Primary structure of CD52. J. Biol. Chem. 270, 6088–6099. 10.1074/jbc.270.11.6088, PMID: 7890742

[ref22] Waks A. G. Winer E. P. (2019). Breast cancer treatment: a review. JAMA 321, 288–300. 10.1001/jama.2018.19323 30667505

[ref23] Winqvist M. Mozaffari F. Palma M. Sylvan S. E. Hansson L. Mellstedt H.. (2017). Phase I-II study of lenalidomide and alemtuzumab in refractory chronic lymphocytic leukemia (CLL): effects on T cells and immune checkpoints. Cancer Immunol. Immunother. 66, 91–102. 10.1007/s00262-016-1922-6, PMID: 27815572 PMC5222940

[ref24] Yang L. Li Y. Bhattacharya A. Zhang Y. (2019). A recombinant human protein targeting HER2 overcomes drug resistance in HER2-positive breast cancer. Sci. Transl. Med. 11:eaav1620. 10.1126/scitranslmed.aav1620, PMID: 30674653 PMC6409101

[ref25] Yu G. Wang L. G. Han Y. He Q. Y. (2012). ClusterProfiler: an R package for comparing biological themes among gene clusters. OMICS 16, 284–287. 10.1089/omi.2011.0118, PMID: 22455463 PMC3339379

[ref26] Zhao Y. Su H. Shen X. Du J. Zhang X. Zhao Y. (2017). The immunological function of CD52 and its targeting in organ transplantation. Inflamm. Res. 66, 571–578. 10.1007/s00011-017-1032-8, PMID: 28283679

[ref27] Zmira O. Halpern A. I. Abraham L. Achiron A. (2020). Efficacy and safety of alemtuzumab treatment in a real-world cohort of patients with multiple sclerosis. Acta Neurol. Belg. 10.1007/s13760-020-01375-6, PMID: [Epub ahead of print] 32447722

